# Foams with Enhanced Ductility and Impact Behavior Based on Polypropylene Composites

**DOI:** 10.3390/polym12040943

**Published:** 2020-04-18

**Authors:** Santiago Muñoz-Pascual, Cristina Saiz-Arroyo, Zina Vuluga, Mihai Cosmin Corobea, Miguel Angel Rodriguez-Perez

**Affiliations:** 1 Cellular Materials Laboratory (CellMat), Universidad de Valladolid, 47011 Valladolid, Spain; marrod@fmc.uva.es; 2 CellMat Technologies S.L., Paseo de Belen 9-A (CTTA Building), 47011 Valladolid, Spain; c.saiz@cellmattechnologies.com; 3 National Institute for Research and Development in Chemistry and Petrochemistry-ICECHIM, 202 Spl. Independentei, 060021 Bucharest, Romania; zvuluga@icechim.ro (Z.V.); mcorobea@yahoo.com (M.C.C.)

**Keywords:** polypropylene, long-chain branching, SEBS, halloysite nanotubes, foam, impact strength, glass fiber, ductility, cellular structure, open cell

## Abstract

In this work, formulations based on composites of a linear polypropylene (L-PP), a long-chain branched polypropylene (LCB-PP), a polypropylene–*graft*–maleic anhydride (PP-MA), a styrene-ethylene-butylene-styrene copolymer (SEBS), glass fibers (GF), and halloysite nanotubes (HNT-QM) have been foamed by using the improved compression molding route (ICM), obtaining relative densities of about 0.62. The combination of the inclusion of elastomer and rigid phases with the use of the LCB-PP led to foams with a better cellular structure, an improved ductility, and considerable values of the elastic modulus. Consequently, the produced foams presented simultaneously an excellent impact performance and a high stiffness with respect to their corresponding solid counterparts.

## 1. Introduction

Polypropylene (PP) is a polymer with relatively low cost and excellent properties such as low density, easy processability, good recyclability, moisture resistance, high thermal stability, or excellent chemical and corrosion resistance. These versatile properties make it one of the most used commodity plastics, and this is the reason why the mechanical performance of PP arouses scientific interest. PP exhibits good stiffness and strength, but its use in certain applications is somehow limited by its impact strength because of its high degree of crystallinity and its high glass transition temperature [1,2,3]. Consequently, it could be said there is a gap between its mechanical properties and that of the engineering plastics, limiting its use for certain applications. 

However, this gap can be adequately filled by applying certain approaches such as the modification of the crystalline structure and the addition of fillers or reinforcements [4]. One option is related with a proper selection of the polymorphism of the crystalline structure in PP (α, β, γ and smectic mesophase can be obtained). It is known that the β-phase of PP outperforms the α-PP in toughness [5]. However, unless highly selective nucleating agents are used, PP typically crystallizes in the α-form [5]. Other strategy used to improve the impact resistance of PP is to blend it with other polymers, especially with elastomers [6]. A large amount of studies have been carried out using different types of elastomeric phases such as poly(styrene–ethylene–butylene–styrene) (SEBS) [2,7], ethylene–propylene rubber (EPR) [8,9], ethylene propylene–diene monomer elastomer (EPDM) [1,10], or poly(ethylene–co–octene) (POE) [11,12,13]. Regardless of the type of elastomer, typically, a significant increase of impact resistance is achieved, however, and as a consequence, stiffness and strength are typically reduced. In these cases, the addition of a rigid organic or inorganic phase can cause a toughening effect without stiffness reduction. For this purpose, many different types of fillers have been successfully employed in combination with elastomers and a PP matrix; the most common are silica [14,15], organoclays [16,17,18,19,20,21,22], or talc [23,24]. So, toughening of PP has been widely reported in literature, using elastomers, rigid particles, and combinations of both.

In the case of foamed parts based on PP, it can be said that the impact resistance is even poorer than in solid PP. A dramatic reduction in impact strength is typically achieved in PP-based foams even at high relative densities. This phenomenon is called ductile-brittle transition [25]. As it is well known, there is a strong dependence of the properties of cellular materials (like elastic modulus, strength or thermal conductivity) with density. In a foamed material, any property can be estimated using the scaling (or power) law models [26]. These empirical relationships relate a certain property of the cellular material ($X_{foam}$) with the same property of the corresponding solid polymer ($X_{solid}$) and its relative density (ratio between the density of the foam (*ρ*_*foam*_) and the density of the corresponding solid (*ρ*_*solid*_)) (Equation (1)):

(1) $$X_{foam}=C\cdot X_{solid}\cdot {\left(\frac{\rho_{foam}}{\rho_{solid}}\right)}^{n}$$

Typically, *C* exhibits values close to 1 and *n* reaches values between *n* = 1 and *n* = 2, depending on the cellular structure and the considered property (for example, elastic modulus). A high value of *n* implies a significant lowering of the foam property when relative density is reduced, which means that if this property is critical for the final use of the item, the foaming of this particular item would not be a good approach.

Throne [25] studied the mechanisms underlying the impact response of structural foams (foams with well-defined solid skins) based on polyphenylene oxide (PPO) and polystyrene (PS), remarking the problem of the ductile-brittle transition appearing even at low levels of foaming (high relative densities). This means that even the most ductile polymers can fail in a brittle manner if they are foamed and subjected to high strain rates. The proposed scale law for the break energy during an impact test is (Equation (2)):

(2) $$\;I_{foam}=I_{solid}{\left(\frac{\rho_{foam}}{\rho_{solid}}\right)}^{m}{\left(\frac{h_{foam}}{h_{solid}}\right)}^{n}$$

where $I_{foam}$ and $I_{solid}$ are the absorbed energies during the impact for the foamed and the unfoamed sample respectively, and $h_{foam}$ and $h_{solid}$ are the thickness of the foam and solid samples respectively.

The first approximation of Throne for thermoplastics was *m* = 4 and *n* = 2. On the other hand, Tejeda et al. [27], denoted that most of the polymers exhibit a performance following trends with values of *m* and *n* close to 2 and 3 respectively, but for PP, the adequate values were *m* = 3 and *n* = 1. The high values reached by *m* in both cases reflect the magnitude of the ductile-brittle transition problem, that limits the application of foams in sectors like automotive or aeronautics in which the plastic parts must fulfill stringent mechanical requirements.

It is important to remark that this model does not take into account the characteristics of cellular structure or the distribution of the solid skins in the case of structural foams. Throne observed that the experimental discrepancies with the data obtained by Equation (2) were caused by the density gradient and the skin-core distribution [25]. Thicker skins increased the impact resistance, the curves being described by the model at the lower bound. 

The influence of cellular structure on the impact resistance of foams has been studied by numerous authors. Some of them have used foaming processes like the solid state foaming to obtain finer structures and to contrast the effect of the cell size. For example, Kumar and Bureau [28] applied it to polycarbonate (PC) structural foams. The results showed that brittle PC can acquire ductility if it is foamed (microcellular foams with cell sizes of around 3–9 μm). This phenomenon appeared as a result of the multiple fracture initiation and growth and the coalescence of voids formed at cells acting as stress concentrators. Rachtanapun et al. studied the microcellular foam batch processing of HDPE/PP blends and the relationship of cellular structure and Izod impact strength [29]. The improvement in the impact strength was achieved for well-developed and uniform microcellular structures with small cell sizes and high cell population densities. On the other hand, Jin-Biao Bao evaluated the tensile and impact behavior of PS microcellular foams with bi-modal cell morphology obtained by batch foaming [30]. Relative impact strengths of bi-modal foams were always higher than those of monomodal ones, in the range of relative density varying from 0.2 to 0.5. Bao [31] also carried out similar experiments using the solid-state batch foaming in order to obtain PP foams with different densities. The impact response was improved for samples with cell sizes lower than 10 μm. 

The same trend was observed by Bledzki [32], using MuCellTM technology. He studied the different types of structural foam that can be produced using PC. A detailed explanation about the influence of the distance between cells on the impact resistance was given: If the separation is small enough, only uniaxial movement is allowed and plastic flow is predominant. On the other hand, triaxial stress favors brittle behavior, as it can occur in solids and non-microcellular foams. In another study, the same author [33] proved that brittle solid samples of PC can be improved by MuCell foaming, and especially using gas counter pressure, obtaining structural foams with cell sizes about 10 µm. However, if the solid material was tough, the inclusion of a cellular structure resulted in lower absorbed energy. Stumpf [34] added organic supramolecular nucleating agents in PP and used MuCell foaming. The cell size was reduced from 120 to 20 µm, improving the Charpy impact absorbed energy, because of the lower cell strut width. 

Michaeli et al. [35] studied the influence of density, skin thickness, density, and cell size using an amorphous polymer as PC and a semi-crystalline polymer as polybutylene terephthalate (PBT). The polymers were saturated with gas in an autoclave using CO_2_ as blowing agent, and then, a conventional injection machine was used to obtain structural foams. Skin thickness was playing a significant role in PC samples, improving the impact response. Higher cell density and lower cell size improved the absorbed energy in both cases. In high density foams, cell density was lower and cells acted more as stress concentrators instead as stress arrestors. Using the same fabrication method, Michaeli [36] tested foamed PP and polycarbonate (PC) samples using instrumented falling weight impact tests (puncture tests). Interesting results were found for PP tested at different speeds. At low speeds, a fine structure (small cell size) led to better results than compact samples. However, at higher strain rates, it was even worse than a coarse structure. Other interesting study was carried out by Zhou [37]. In his study, the addition of a nucleating agent modified the cellular structure in injection molded PP foams, being characterized by smaller cell size, higher cell density, and a more homogeneous distribution of cells. The improvement of cellular morphology led to a better impact response.

So, as the previous studies note, the morphology and characteristics of the cellular structure play a crucial role in the impact response of foams. An improvement in cellular structure, reached by creating more homogeneous cell size distributions and with low values of cell size could translate into an enhancement of the impact response and in avoiding or diminishing the effects associated to the ductile-brittle transition appearing in PP foams. 

If the improvement of the impact resistance is the key objective, it seems logical that the approaches used in the solids should also be applied for foams. For example, the use of fillers is considered in the study carried out by Spoerrer et al. [38]. A lower reduction of the impact resistance with respect to that displayed by unfilled PP foams was achieved for talc-filled PP foams and especially for glass fiber filled foams. The use of polymer blends and elastomers is another solution that foams have inherited from solids. In the publication of Estrada-Núñez et al. [39], foams based on blends of high density polyethylene (HDPE) and PP were produced using injection moulding with azodicarbonamide as blowing agent. The use of an elastomer (SBS) as compatibilizer raised the impact resistance of foams, and in particular, for high concentrations of PP, foamed blends were similar to their unfoamed counterparts.

Finally, a recent novel approach to the problem (using injection moulding with chemical blowing agent) is given by Gong et al. [40] and Muñoz-Pascual [41]. Gong et al. [39] obtained foamed PP/POE blends. The impact resistance obtained for the foams before the brittle-ductile transition (due to the presence of the elastomeric like phase) was higher than for the solids. This is a behavior that is repeated at low temperatures. When the POE content was increased (or the temperature was higher), the unfoamed PP/POE was better. Muñoz-Pascual [41] obtained ductile foams and analyzed the differences between processing conditions. The differences in the cellular, crystalline, and elastomer morphology determined the behavior of solids and foams. It was concluded that high mould temperatures favored the impact resistance of solids and low mould temperatures improved the absorbed energy in foams. 

Bearing all the previous ideas in mind, it can be said that the two approaches can be considered to improve the impact response of cellular materials: the toughening of the polymeric matrix and/or the improvement of the quality of cellular structure (with the main target of reducing *m* values in Equation (2)). 

The objective of this work is to improve the impact behavior and the ductility of PP-based foams. For this purpose, the two strategies mentioned above have been combined. On the one hand, modifications including the addition of elastomeric and rigid phases would be aimed at improving the properties of the base PP matrix. On the other hand, as the simple inclusion of additional phases is not enough because of the apparition of the ductile-brittle transition and the important role played by the cellular structure, formulations with higher foamability levels have been considered to be able to generate foams with optimized cellular structures.

An easy and straightforward way to improve the cellular structure of PP foams is the use of long-chain branched polypropylene (LCB-PP). The low melt strength and low extensional viscosity of linear PP grades typically lead to a rupture of the cell walls under the elongational forces occurring during cell growth which typically results in foams with larger cell sizes and heterogenous cellular structures. LCB-PP grades exhibit a strain hardening behavior that is closely related to a higher melt strength [42,43]. For this reason, the use of LCB-PP could be an effective way to improve the cellular structure, and then, to raise the overall mechanical performance of the materials. Using this type of PP, interesting results have been obtained. Wang [44] studied the tensile properties in LCB-PP foams produced by core-back injection molding. The addition of a nucleating agent reduced the cell size and the tensile toughness and ductility increased in comparison with the non-nucleated foams.

Multiphasic foams comprising all the previous mentioned phases (elastomers, rigid particles, and a PP grade with high foamability levels) have been produced using the so-called improved compression molding route (ICM). The effectiveness of the employed approaches will be evaluated by analyzing the obtained foams and their corresponding solids. Tensile and impact tests (puncture and Izod tests) were performed to understand how the changes in the formulation affect the mechanical performance of each particular formulation. The results have been correlated with the cellular structure of the materials.

## 2. Materials and Methods

### 2.1. Materials

As polymeric matrices, a heterophasic polypropylene copolymer (L-PP) and a long branched polypropylene random copolymer were employed (LCB-PP). The linear polypropylene used for the study was BJ380MO, a low viscosity polypropylene manufactured by Borealis AG (Vienna, Austria) and presents a melt flow index (MFI) of 80 g/10 min (230 °C/2.16 kg) and a density of 0.906 g/cm^3^. The grade of long chain branched polypropylene employed for the study was Daploy WB 260 HMS, a random polypropylene-polyethylene copolymer that was provided by Borealis AG and having a MFI of 2.4 g/10 min (230 °C/2.16 kg). Kraton 1652 G (SEBS) from Kraton Polymers (Houston, TX) is a linear poly[styrene-(ethylene-co-butylene)-styrene] block copolymer with a styrene content of 29%, number average molecular weight (Mn) of 79.100, density of 0.91 g/cm^3^, and MFI = 5.00 g/10 min (230 °C/5 kg) and was used as an impact modifier. Treated Halloysite nanotubes (HNT-QM), a white color powder with kaolin content >95%, trade secret <5%, and quartz <1%, produced by ABC Company (Mumbai, India) and provided by Aalborg University (Denmark) was used as inorganic nanofiller. Maleic anhydride-grafted polypropylene (MAPP), Polybond 3200 from Crompton (Middlebury, CT, USA) with a density of 0.91 g/cm^3^, a melting point of 157 °C was used as a compatibilizer agent. Strands of chopped glass fibers with 636 sizing, coated with silane, ThermoFlow 636 (GF), produced by Johns Manville (Denver, CO, USA), were used as reinforcing agent and were provided by FPK Lightweight Technologies S.Coop (Peine, Germany).

The selected chemical blowing agent was azodicarbonamide Porofor ADC/M-C1, (supplied by Lanxess, Colonia, Germany), with an average particle size of 3.9 µm and a density of 1650 kg/m^3^. The decomposition temperature is 210 °C and the volumetric gas yield at this temperature is 228 mL/g. Antioxidants Irgafos 168 (from Ciba, Basel, Switzerland) in a proportion of 0.08 wt. % and Irganox 1010 (from Ciba) in a proportion of 0.02 wt. % were added to all formuations to prevent thermal oxidation of the polymer.

### 2.2. Preparation of PP Composites

The PP composites were obtained in dynamical conditions using a Leistritz LSM 30.34 corotating twin-screw extruder at a screw speed of 220 rpm. Prior, masterbatches based on SEBS, HNT-QM and MAPP were prepared. PPBJ, masterbatch, and GF were mixed in a rotating mixer at room temperature for 30 min. Extruder temperature profile from hopper to die was 180, 185, 190, 195, 200, 210, 205, 200, 170, and 160 °C, respectively. The extruded filaments passed through the cooling water bath and were granulated with a Leistritz Pelletizer.

The chemical composition of the considered formulations is summarized in Table 1. As it can be seen, the variation in terms of chemical composition between the different considered samples is centered in the concentration of linear and LCB polypropylenes. So, the amount of MAPP, SEBS, HNT-QM, and GF remains constant for all the developed formulations. By considering such formulations it is possible to evaluate the effect of the presence of a polymer with good foamability levels on the mechanical behavior and foam structure.

Extruded pellets were employed to produce two different types of thermoformed solid samples: Rectangular plaques of 155 mm length, 75 mm width, and 4 mm thickness and discs of 150 mm in diameter and 2 mm in thickness. All the solid specimens were produced by using compression molding and a two-hot plate press. A temperature of 175 °C and pressure values of 17.8 MPa for plaques and 10.4 MPa for discs were employed. The rectangular plaques were cut with the appropriate form and subsequently used to perform tensile tests. From the 2 mm thick discs, three samples of 60 mm in diameter were extracted to perform the instrumented falling weight impact tests.

### 2.3. Foaming Process

To obtain foamed specimens, the improved compression moulding (ICM) route was used [43]. First, the formulations summarized in Table 1 were blended with a chemical blowing agent (azodicarbonamide) by melt compounding using a twin-screw extruder (Collin Teach Line model ZK 25T SCD 15) and a temperature profile of 140, 145, 150, 155, and 160 °C (from the feeding zone to the die). The processing temperatures were selected to avoid premature decomposition of the blowing agent. The screw speed was 45 rpm. The concentration of azodicarbonamide was fixed for all the formulations at a constant value of 1 wt. %.

Using the so-obtained materials, cylindrical-shaped thermoformed solid precursors of 2 mm in height and 150 mm in diameter were produced using a two hot-plates press set at a temperature of 165 °C. A pressure of 10.4 MPa was applied during 5 min.

**Figure 1:**
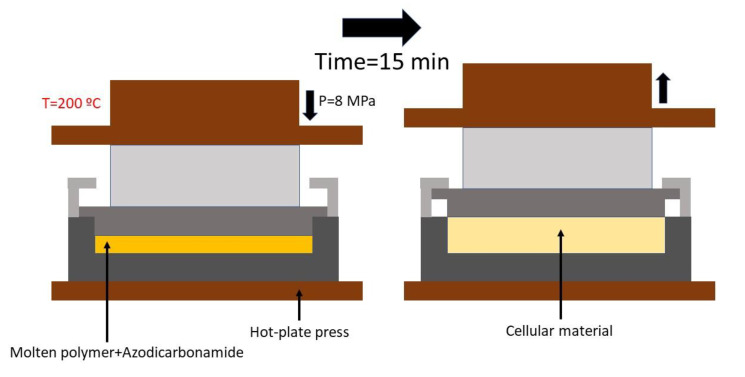
Improved compression molding method schema with the used parameters in these experiments.

These cylindrical precursors were introduced into a special mold that can control the final density of the material [45]. The mold is subjected to both temperature and pressure in a two-hot plates press. A pressure of 8 MPa and 200 °C was applied for 15 min. After this time, when azodicarbonamide was fully decomposed, pressure inside the mold stabilized. At this point, the applied pressure was relaxed allowing the polymer to expand. The depressurization rate was 1.3 MPa/s. The mold was cooled down using cold water to stabilize the cellular structure as fast as possible. The relative density of all the produced foams was fixed in a value of around 0.62 (corresponding to an expansion ratio of 1.61). A schema of the production method with the used pressure, time, and temperature parameters is shown in Figure 1.

### 2.4. Characterization

The density of the solid and foamed materials was measured using the geometric method; this is by dividing the weight of each specimen between its corresponding volume (ASTM Standard D1622-03). Four specimens were measured to obtain an average value. Relative density was obtained as the density of the foams divided by the density of the solid precursor from which the foam was produced. The open-cell measurements were performed with a gas pycnometer Accupyc II 1340 from Micromeritics, according to ASTM D6226-10 [46]. Four prismatic pieces of each material were measured. 

Scanning electron microscopy (SEM) was used to analyze the cellular structure of the foamed specimens. The micrographs were taken using a Jeol JSM-820 scanning electron microscope. The samples were cooled down with liquid nitrogen and then fractured. A thinner layer of gold was sputtered on the fractured surface to make it conductive. Average cell diameter was determined using an image-processing tool based on ImageJ software [47]. Relevant statistical parameters such as the standard deviation (SD) of the cellular structure were calculated according to Equation (3).

(3) $$SD=\sqrt{\overset{n}{\underset{i=1}{\sum }}\frac{{\left(\phi_{i}-\phi \right)}^{2}}{n}}$$

where *n* is the number of counted cells, $\phi_{i}$ is the cell diameter of cell I, and ϕ is the average diameter of the cells. This parameter accounts for the width of the cell size distribution. In order to obtain a relative measure of the homogeneity of the cell size distribution, *SD/ϕ* parameter was used, defined as the standard deviation divided by the average cell size. A low value of this parameter, *SD/ϕ*, indicates a more homogeneous cellular structure. 

Cell density ($N_{v}$) was determined using Kumar’s method [48] according to Equation (4), were *A* is the analyzed area and *n* is the number of cells in that area.

(4) $$N_{v}={\left(\frac{n}{A}\right)}^{\frac{3}{2}}$$

Tensile properties of the samples were evaluated at room temperature using an Instron machine model 5500R6025, using the standard ISO 527. Elastic modulus was determined at strain rate of 2 mm/min. Stresses and elongations at yield point and at break were obtained at strain rate of 50 mm/min. The samples were stored at 23 ± 2 °C and 50 ± 10% relative humidity for 24 h before each measurement. The experiments were performed under the same conditions. Two samples of each reference were tested.

The notched Izod impact strength of the specimens was tested using a Frank 53566 Izod pendulum according to the UNE-EN ISO 180/A standard. The experiments were carried out at room temperature, and the average value was obtained from over five specimens for each condition.

Instrumented falling weight impact tests (IFWI) were performed at room temperature using an impact tester designed by CellMat Laboratory and built by the company Microtest. The striker is a hemispherical tipped dart of 20 mm in diameter. The force transducer (KISTLER type 9333A) was located in the upper part of the striker and the force data acquisition frequency was 55.3 kHz. The displacement of the striker was measured by using a laser triangulation sensor, LDS90/40, from LMI Sensors-95. The incident energy was 55.66 J (*m* = 5.75 kg, *v* = 4.4 m/s).

The experiments and the analysis of data were carried out following the procedure described in ISO 6603 standard. In this study, the magnitude of interest, as a measure of the samples ductility, was the perforation energy, which is defined as the energy employed to reach the deflection at which the force is half of the maximum force (perforation deflection). The samples were stored at 23 ± 2 °C and 50 ± 10% relative humidity for 24 h before testing them and the experiments were carried out under the same conditions. Three samples of each material were tested to obtain the average response. The mean thickness for solid materials was 2 mm, whereas the thickness of foamed materials was about 3 mm. To eliminate the possible effects associated to the differences in terms of thickness between the solids and the foams, the perforation energy values were normalized to the squared thickness of the sample. 

To evaluate the influence of the cellular structure on foam properties, the relative properties (foam property divided by the property of the corresponding solid material) have been studied as function of the relative density. This allowed us to compare the obtained data with the expected trends for foams expressed by Equation (1) taking into account that, in general, most of the mechanical properties of foams descend with the square of relative density [25]. To obtain the *n* values, a fitting to a power equation was carried out. Evaluating the values obtained for *n* and for each of the analyzed properties was helpful to evaluate the level of effectiveness of the strategies employed to improve the materials.

## 3. Results and Discussion

### 3.1. Cellular Structure

**Figure 2:**
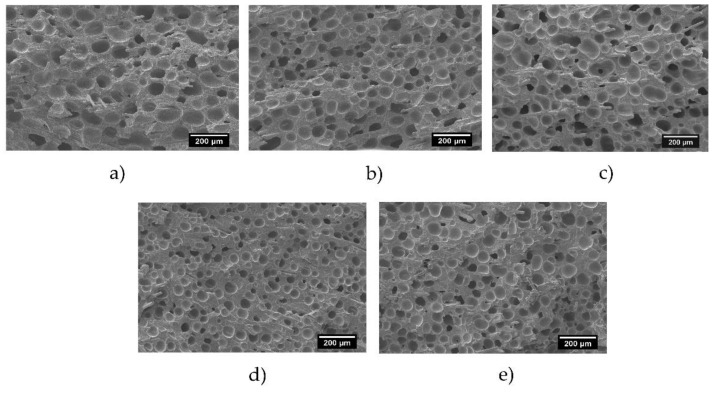
Scanning electron microscopy (SEM) micrographs of several obtained samples: (**a**) long-chain branched polypropylene (LCB-PP) 0% (**b**) LCB-PP 25% (**c**) LCB-PP 50% (**d**) LCB-PP 75% (**e**) LCB-PP 100%. The scale bar represents 200 μm.

Figure 2 shows the examples of micrographs of the foams produced. In all cases, isotropic and homogenous cellular structures were obtained.

**Figure 3:**
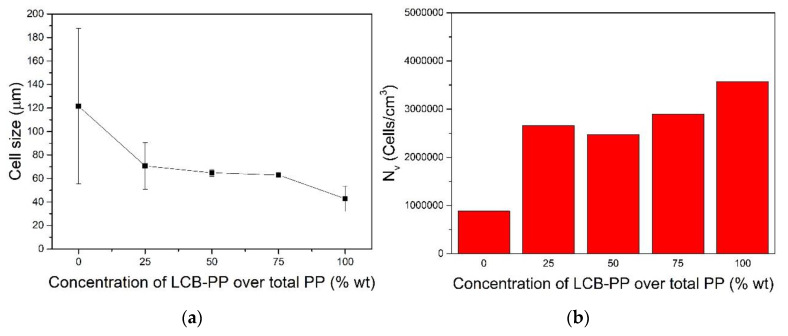
(**a**) Cell size and (**b**) cell density as a function of LCB-PP concentration.

As it can be appreciated in Figure 3, cell size was reduced with the increment on the concentration of LCB-PP and the cell density was increased. This effect is typically associated to the addition of LCB-PP. This polymer, specially designed for foaming applications presents a higher melt strength than linear polypropylene and this typically translates into the generation of cellular structures with better quality, (smaller cell sizes, lower open cell content and/or narrower cell size distributions) [42]. The reduction in terms of cell size was not homogeneous; with the addition of 25% LCB-PP the cell size was reduced about 42%. Then, between the 25% LCB-PP and the 75% LCB-PP compounds, the cell size was only reduced about 11%. So, a small plateau was reached for this magnitude with the variation of the concentration of the PP with high foamability. As it will be observed later, this could be determinant in the mechanical performance of the materials.

On the other hand, the quality of the cellular structure is not only measured in terms of cell size. An important parameter such as the homogeneity of the cellular structure can be quantified using the standard deviation of the cell size distribution, (Equation (3)). The values for this standard deviation have been presented in Figure 3 as error bars. It can be seen that this standard deviation is clearly reduced (homogeneity of the cell size distribution is improved) when the content of LCB-PP is increased. 

**Figure 4:**
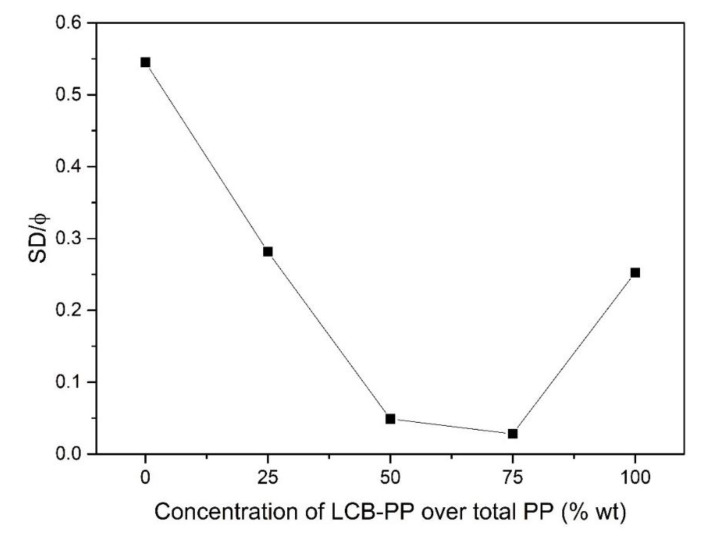
*SD/ϕ* parameter as a function of LCB-PP concentration.

For this case, the high variation of cell size between the different materials (121 μm in the higher case and 43 μm in the lower: almost three times lower) makes more appropriate the use of *SD/ϕ* parameter to compare between them. As can be seen in Figure 4, there is a clear reduction (20% reduction) of this parameter when foams with LCB-PP contents higher than 50% are produced. This indicates that the addition of significant amounts of LCB-PP is also accompanied by the generation of narrower (more homogeneous) cell size distributions, as it was to be expected, because of the higher melt strength of this kind of polypropylene.

**Figure 5:**
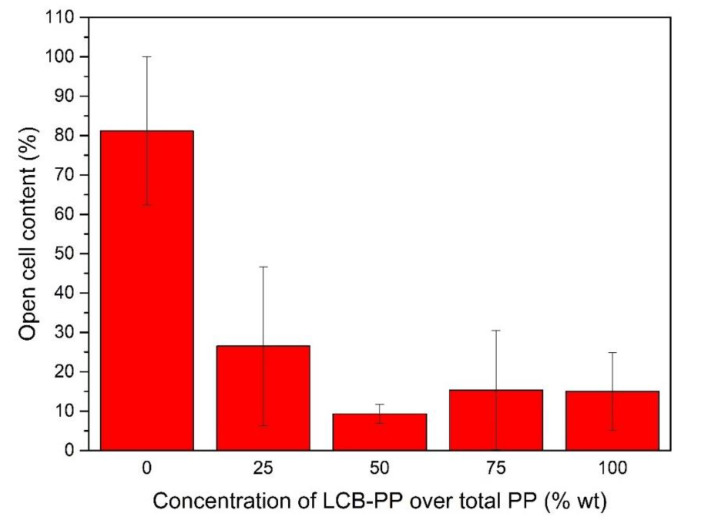
Opel cell content of the foams as a function of LCB-PP content.

The open cell content was measured for the different compounds, obtaining the following graph (Figure 5). As can be seen, a higher open cell content was observed for L-PP. This was expected: the low strain hardening of L-PP led to a higher cell rupture process. This was seen by Laguna-Gutierrez [46]. According to LCB-PP increment, the open cell content was sensibly lower reaching a minimum value for the 50% LCB-PP foam. This parameter was directly related with mechanical properties as Laguna-Gutierrez observed [49]. The compound with the lower value (50% LCB-PP) also showed the minimum *SD/ϕ* value in Figure 3, being the material with better cellular structure.

### 3.2. Mechanical Properties: Tensile Tests

The tensile properties of the two polypropylene matrices (L-PP and LCB-PP) were measured following the procedure described in the previous section and the experimental data are collected in the following table (Table 2):

This information has been used to help understand the behavior of the composite blends prepared in this paper. The L-PP grade is characterized by presenting a high stiffness and a low ductility, whereas, the LCB-PP elastic modulus is much lower (around the half). The counterpart is that the ductility of the last is around five times higher. So, a stiff L-PP and a ductile LCB-PP were used, being the mechanical properties of these materials crucial to determine the mechanical properties of the blends. It is supposed that, in a first approximation, if these polymers are blended, the properties of the obtained composite are contained between the properties of both, proportionally to the volume fraction, following the next equation:

(5) $$E=V_{f}E_{1}+\left(1-V_{f}\right)E_{2}$$

where, E, $E_{1}$, and $E_{1}$ are the modulus of the blend, the first component and the second, respectively, and $V_{f}$, the volume fraction of the first component.

Figure 5 displays the results corresponding to the tensile elastic modulus of the produced blends, both the raw experimental values and the relative ones, with the n-exponent of the scale model representation (Equation (1)). Moreover, the theoretical modulus of L-PP+LCB-PP blends using Equation (4) are represented for the shake of comparison: 

The results showed that the substitution of L-PP by LCB-PP induced a reduction of the stiffness of the solid samples, as can be seen when it is compared with the theoretical value of the raw material combination. The maximum level of reduction was achieved for the material not containing L-PP and it was of about 38%. This reduction can be explained taking into account the differences in elastic modulus between the two PP grades. Under the same preparation and testing conditions, the elastic modulus of the utilized L-PP was 1528 MPa while for LCB-PP was 852 MPa. The theoretical elastic modulus of the LCB-PP is about 44% lower than that of the L-PP. This ratio of variation is very close to the one achieved between the two extreme samples. On the other hand, the experimental data shows that the addition of glass fiber-treated halloysite nanotubes countered the supposed reduction of stiffness derived from the addition of nearly 20% wt of elastomer (SEBS) because for all the solid blends the experimental data of the composite blends are similar to the one obtained for the pure blends. 

However, the elastic modulus of the foams seems to be almost constant and does not show any clear trend with the variation in the concentration of LCB-PP. Indeed, if the trend obtained for the solids would be transferred to the foams, the modulus of the foam with the higher content of LCB-PP should present an elastic modulus of around 300 MPa. However, the obtained value was only 12% lower than the one corresponding to the material without LCB-PP, this is about 400 MPa. Besides that, the values of the elastic modulus of the foams appear to be independent of the concentration of LCB-PP. This result could indicate that the changes induced in the cellular structure of the composite foams by the increase in the concentration of LCB-PP are compensating the decrease of the mechanical properties of the solid polymeric matrix comprising the foams, giving as a result foams with similar levels of stiffness for all the produced blends. 

**Figure 6:**
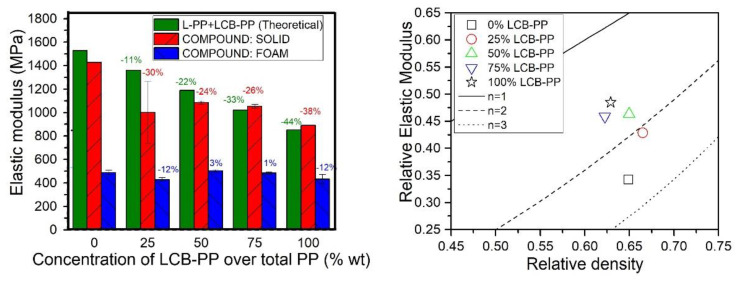
Tensile elastic modulus of solids samples and foams (**left**) and relative elastic modulus of foams in the scale model representation (**right**). The theoretical elastic modulus of a blend of L-PP and LCB-PP using the model expressed in Equation (4) is represented in the left graph. The percentages in the left graph indicated the percentage of variation of the elastic modulus of each sample with respect to the 0% LCB-PP compound value, for solids (**red**) and foams (**blue**).

As all the foamed materials present similar values of the tensile elastic modulus, the loss of stiffness in solids when the LCB-PP content was incremented causes the improvement of relative elastic modulus, as can be seen in the relative property representation in Figure 6 (right). Values of *n* lower than 2 are achieved for materials containing contents of LCB-PP higher than 50%, being the maximum the composite including a 100 % of LCB-PP. This means that the higher content of LCB-PP of the foam, the lower the loss of properties with respect to the solid materials in terms of stiffness.

**Figure 7:**
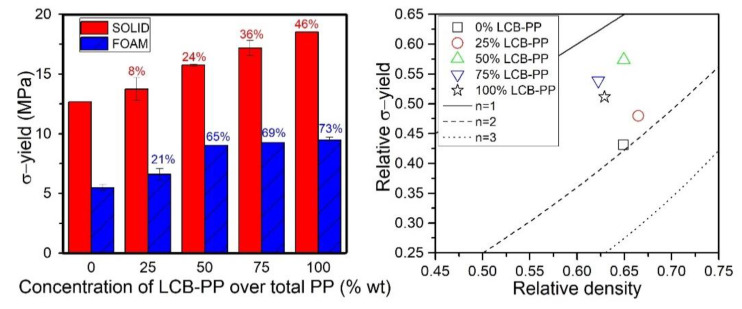
Stress at yield of solids samples and foams (**left**) and relative stress at yield of foams in the scale model representation (**right**). The percentages in the left graph indicate the percentage of improvement with respect to the 0% LCB-PP compound value, for solids (**red**) and foams (**blue**).

The tensile resistance of the foams and solids was analyzed studying the tensile strength (at yield point). Figure 7 shows the experimental values of stress at yield accompanied by the relative values in the scale model using Equation (1).

It is observed that the inclusion of the LCB-PP, apart from the improvements at the microstructural level in foamed specimens, also resulted in a significant improvement of the stress at yield in the solid specimens However, unlike the elastic modulus, the addition of fibers and elastomer reduced considerably the strength with respect to the neat PP grades used for the study (see Table 2). For the pure polymer the stress at yield was 23.87 for L-PP and 25.06 for LCB-PP, both values higher than the ones measured for the composite blends including fibers and an elastomeric phase. 

The differences between the two pure used PPs was low (around a 5%), however, the gap displayed by the solid materials including 0 and 100% of LCB-PP is significant and about 46% higher in the one containing just the polypropylene grade specifically designed for foaming applications. Consequently, it seems that the strength of L-PP was affected in a higher extend than that of the LCB-PP when fibers and elastomer were added to the system. 

For the foamed specimens, it is observed a certain level of improvement for LCB-PP concentrations equal to or higher than a 50 wt. %. The percentage of increment barely varies when incrementing the concentration of LCB-PP up to a 100 %. Nevertheless, the foamed specimens display higher level of enhancement in terms of strength, due to the presence of the LCB-PP, than their corresponding solid counterparts. It could be said that synergistic effects are accomplished because of the combination of foaming and the different modifications exerted over the different PP grades. The relative properties (Figure 7b) are clearly better for the materials with contents of LCB-PP higher than 50%, which seems to be related to the improved cellular structured observed for these materials (Figure 3, Figure 4, and Figure 5). Moreover, the best relative foam (50% LCB-PP) was the one with lower open cell, strongly setting a relation between mechanical properties and cellular structure. 

**Figure 8:**
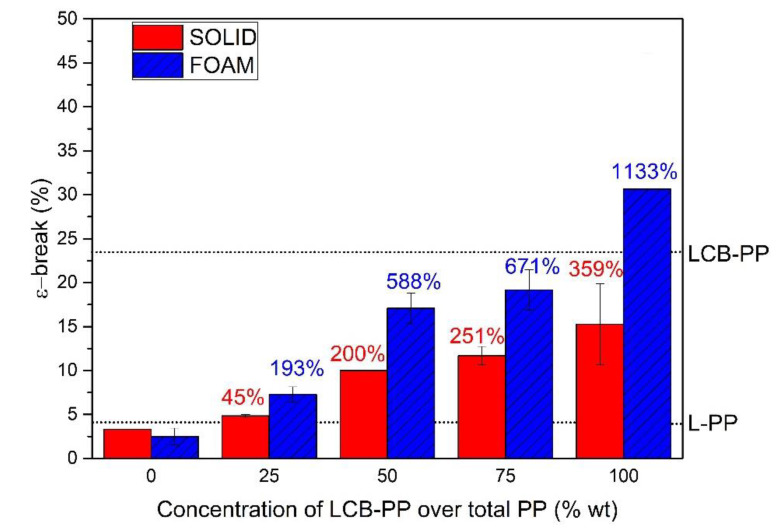
Strain at break of solids samples and foams. The strain at break of the raw used polypropylene is indicated by dotted lines. The percentages in the right graph indicated the percentage of improvement of this sample with respect to the 0% LCB-PP compound value, for solids (**red**) and foams (**blue**).

Similarly, the values of strain at break have been also determined and the experimental results are shown in Figure 8.

In the case of elongation at break, the increment achieved for the systems under study is remarkable: an increment in solids with the percentage of LCB-PP in the compound was achieved. In the two raw used PP (Table 2), LCB-PP elongation was more than five times higher than that of L-PP, the same ratio is achieved between the 0% LCB-PP sample and the 100% LCB-PP sample. Then, the cause of the increment of break strain in solids with the addition of LCB-PP can be directly attributable to the mechanical properties of the used raw PP. Moreover, values of the raw used PP were slightly higher than that of the corresponding compounds, so the addition of elastomer phases, that should cause a strong increment of ductility, were compensated by the addition of rigid fibers. 

On the other hand, foams showed a surprising result regarding this magnitude. As the amount of LCB-PP increases not only the strain increases but also the strain was higher than that associated to the solid material and theses differences became more pronounced as the concentration of LCB-PP increases. For example, the strain for the 0% LCB-PP foam was 25% lower than the solid, for 25% LCB-PP, the strain of foam was already 50% higher than the solid, and so on until the 100% LCB-PP foam, that duplicate the value obtained for the corresponding solid sample. The result was that the total substitution of L-PP by LCB-PP produced more than a 1000% improvement in the strain at break point in foams. This is a striking result, because, typically when a cellular structure is generated, a weakening of the material occurred by the simple fact that polymer is substituted by air. It seems that the combination of elastomeric phases, LCB-PP and a homogeneous cellular structure has a strong synergic effect over the deformation capability, as can be seen in foams. A similar case of superductility was seen by Sun [50] in PP/HDPE foams. In his study, the cavitation of the secondary phase caused the conection and fibrilation of the pores. In this mechanism, the reduced cell size plays a key role. In the PP/HDPE system, the importance of the uniformity of the cellular structure in the deformation mechanism was also studied in depth by Zhou in several articles [51,52,53]. So, it is clear that the inclusion of the LCB-PP combined with foaming in our complex blend led to the formation of foams with a more ductile character. 

The outstanding values of deformation achieved for foams could anticipate a good impact behavior. This was studied in the following section.

### 3.3. Mechanical Properties: Impact Tests

**Figure 9:**
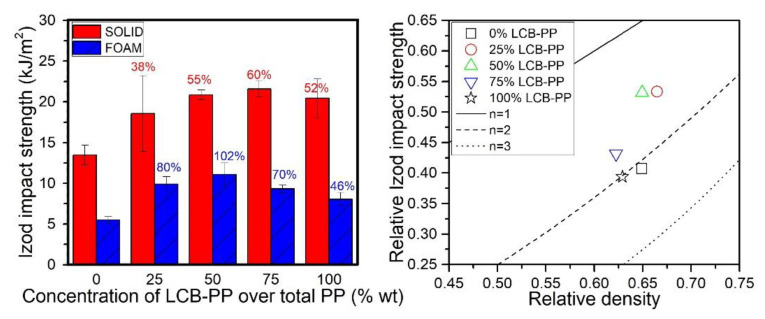
Izod impact strength of solids samples and foams (**left**) and relative Izod impact strength of foams in the scale model representation (**right**). The percentages in the left graph indicates the percentage of improvement of this sample with respect to the 0% LCB-PP compound value, for solids (**red**) and foams (**blue**).

The values of the impact strength obtained in Izod impact tests are presented in Figure 9. As it can be observed, the Izod impact strength of the solids was increased when LCB-PP was added until reaching a maximum for the material including a 75% of LCB-PP, being a 3% and 6% higher than the 50% LCB-PP and 100% LCB-PP compounds respectively. The solid sample containing solely the L-PP, presented the lowest impact resistance and it was improved by about 60% when the adequate percentage of LCB-PP was added. Focusing on foams, their impact resistance was, regardless the concentration of LCB-PP and, as it could be expected, lower than the one obtained for the solids. The trend was similar than that followed in the solids, but the maximum was reached for a concentration of 50% LCB-PP. The achieved improvement was higher than that obtained in solids, because the absorbed energy by the 0% LCB-PP samples was duplicated for the sample with 50% of LCB-PP over total PP. As was seen in previous sections, this was the foam with lower open cell content. 

Paying attention to the scale model representation (Figure 9-right), the obtained values could be considered good results because the usual values followed by impact resistance related parameters in this type of representations for foams tend to be significantly worse, following the trend *n* = 4 [25,40], instead like in this case, where the best foams follow trends with *n* lower than 2. The foams with an intermediate level of LCB-PP were closer to the respective solid property, compared with the extreme compositions.

**Figure 10:**
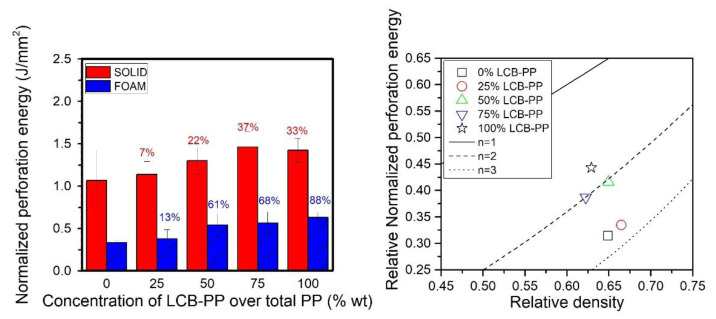
Instrumented falling weight impact (IFWI) normalized perforation energy of solids samples and foams (**left**) and relative IFWI normalized perforation energy of foams in the scale model representation (**right**). The percentages in the left graph indicate the percentage of improvement of this sample with respect to the 0% LCB-PP compound value, for solids (**red**) and foams (**blue**).

The result for perforation energy in IFWI tests are presented in Figure 10.

As it has been seen in other properties, the higher ductility of the LCB-PP produced an increment of the absorbed energy when it is present in the compound. For solid samples, the maximum value was raised for the maximum of LCB-PP up to about 37%.

For foams, the results obtained are remarkable, not for the achieved absolute values but for the extremely good relative property that they all exhibit. The absorbed energy by the foams was increased with the addition of LCB-PP in a higher percentage than the solids (the 100% LCB-PP foam was 88% better than 0% LCB-PP foam). The enhancement of cellular structure seems to play a key role in the improvement of the impact resistance of the foams. The evidence of this reasoning can be observed in the scale model in Figure 10-right: the exponent in the scaling law for these materials is close to the trend *n* = 2, and it is lower for formulations with a higher content of LCB-PP (higher than 50%).

### 3.4. Overall View

**Figure 11:**
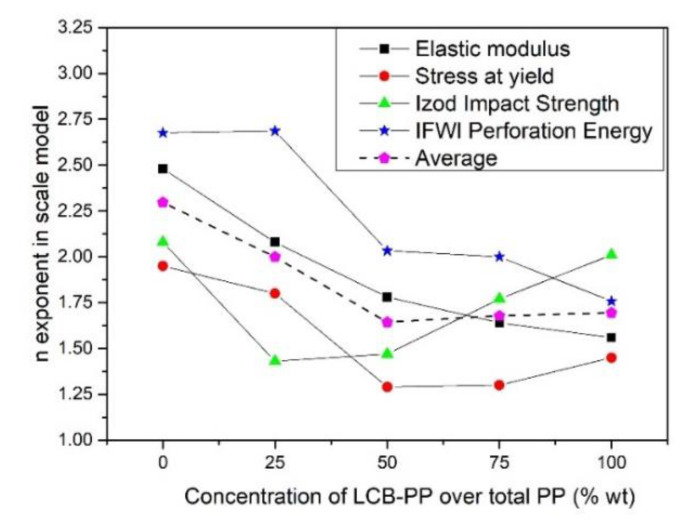
*n* exponent of each property, following the scale model (Equation (1)). The average is also represented.

The *n* exponents of the scale models (Equation (1)) corresponding to the different magnitude and their average have been gathered in the following figure (Figure 11).

Two important facts can be inferred from Figure 11:

- An overall reduction of the values of exponent *n* when the LCB-PP content present in the samples was increased. Achieving a better behavior in the foams than in the solids when the material comprising the cell walls is the same as in the solid samples can only be explained taking into account the changes achieved with regard to the morphology of the cellular structure. Moreover, in Figure 3, Figure 4, and Figure 5, it could be observed that the cell size was reduced, the homogeneity of cellular structure was improved, and the open cell content was reduced until 50% LCB-PP compound, and then, the trend smoothed. This trend seems to be correlated with the plateau (or slightly worsening) that is reached for some magnitudes for LCB-PP contents higher than 50%, as can be seen in the average of the *n* exponents. Moreover, the 50% LCB-PP compound was characterized by its low open cell content, that following the literature [49] could be the reason for its good properties. This implies that there is a determinate percentage for which an increment of LCB-PP does not improve the cellular structure (and then, the mechanical properties). This have been seen previously by Laguna-Gutierrez, in pure blends of L-PP and LCB-PP, obtained also by improved compression molding [43].
- The low values of *n* obtained for impact properties, especially for IFWI, being the expected values near to 4. A good explanation for the outstanding values of impact absorbed energy could be based on the outstanding values of elongation at break. Some studies have shown that a poor behavior at falling weight impact tests was due to the reduction in elongation at break [54]. In our case, the elongation was not reduced when foaming, in fact, it was increased and this increment in ductility could lead to an increment in the toughness of the foams. This increment of elongation at break in foams when the LCB-PP content was raised to values above to those achieved for the solids with the same composition can be also explained by an optimized quality of the cellular structure [51,52,53].

It becomes clear that the modification in the cellular structure induced in the foamed specimens because of the presence of the LCB-PP are responsible for the very high levels of improvement achieved in the foamed samples.

## 4. Conclusions

Different formulations containing elastomeric polymers (SEBS) and rigid particles (GF and HNT) were foamed to a density reduction of 38% using the improved compression moulding (ICM) route, substituting different amounts of a linear polypropylene by a long-chain branched polypropylene (LCB-PP). As it was expected, because of the better foamability of this kind of polymer, the cellular structure of foams showed changes with the inclusion of LCB-PP; a reduction of the average cell size, a narrower cell size distribution, and a lower cell size were achieved. 

The mechanical properties of the compounds differing in the LCB-PP/L-PP content were measured using tensile and impact tests. Because of the lower modulus of LCB-PP with respect to L-PP, a reduction in the elastic modulus was observed for solids with the increment of LCB-PP concentration. However, the modulus in foams was constant because of the enhancement of cellular structure. Regarding the stress at yield, the presence of LCB-PP increased the value of the solids, but no further improvements were achieved for concentrations higher than 50 wt. % of LCB-PP in foams. On the other hand, outstanding values for elongation at break were reached for foams including LCB-PP in their composition. Moreover, at concentrations higher than 25 wt. % they exceeded the performance of the solids. Synergic effects between the use of LCB-PP and the elastomeric phase over the cellular structure could be the cause of this superductile behavior. Finally, Izod impact strength improved for both solids and foams and remarkable values of absorbed energy in IFWI test were obtained. Values near *n* = 1.5 in the relation between relative perforation energy and relative density were found in foams with higher LCB-PP content. The high values of elongation at break seems to be the source of this result.

In conclusion, ductile, tough, and moderately rigid foams have been achieved replacing the linear polypropylene by long-chain branched polypropylene in polypropylene composites that includes both rigid and elastomeric particles. 

## Figures and Tables

**Table 1:** Nomenclature and chemical composition of the considered formulations.

Sample	L-PP (% wt)	LCB-PP (% wt)	MAPP (% wt)	SEBS (% wt)	HNT-QM (% wt)	GF (% wt)
0% LCB-PP	56.50	0.00	2.5	20	1	20
25% LCB-PP	43.37	14.13	2.5	20	1	20
50% LCB-PP	28.25	28.25	2.5	20	1	20
75% LCB-PP	14.13	43.37	2.5	20	1	20
100% LCB-PP	0.00	56.50	2.5	20	1	20

**Table 2:** Tensile properties of pure L-PP and LCB-PP thermoformed samples.

Sample	Elastic Modulus (MPa)	Stress at Yield (MPa)	Strain at Break (%)
L-PP	1528 ± 42	23.87 ± 0.26	4.29 ± 0.25
LCB-PP	852 ± 21	25.06 ± 0.35	22.87 ± 14.41
